# Morpho-anatomical differences among mycoheterotrophic *Afrothismia* spp. (Thismiaceae) indicate an evolutionary progression towards improved mycorrhizal benefit

**DOI:** 10.1007/s00572-020-00951-1

**Published:** 2020-05-08

**Authors:** Stephan Imhof, Benjamin Feller, Anna Heser

**Affiliations:** Spezielle Botanik, Fachbereich Biologie, Philipps-Universität, D-35032 Marburg, Germany

**Keywords:** *Afrothismia*, Mycoheterotrophy, Arbuscular mycorrhiza, Mycorrhizal benefit, Colonization pattern, Evolutionary progression

## Abstract

Achlorophyllous, mycoheterotrophic plants depend on their mycorrhizal fungi for 100% of their carbon supply. Hence, there is strong evolutionary pressure towards a well-organized functioning of the association from the plant’s perspective. Members of the mycoheterotrophic genus *Afrothismia* have evolved elaborate fungal colonization patterns allowing a sustained benefit from external fungal penetration events. On the basis of anatomical details of the root-shoot systems of *A*. *korupensis* and *A*. *hydra*, we elucidate an evolutionary progression between the comparatively simple mycorrhizal pattern in *A. gesnerioides* and the so far most complex mycorrhiza in *A. saingei*. We detected two major advancements: (1) two species, *A. korupensis* and *A. saingei*, use the fungus itself as energy storage, replacing starch depositions used by *A. gesnerioides* and *A. hydra*, and (2) the morphological complexity of hyphal forms in plant tissue compartments increases from *A. gesnerioides* to *A. saingei*. We discuss the omitting of starch metabolism as well as the morpho-anatomical differences as an evolutionary fine-tuning of the compartmented mycorrhizal organization in *Afrothismia*. Our results support the idea of a taxonomic distinction between *Afrothismia* and other Thismiaceae.

## Introduction

Mycoheterotrophic plants (MHP; Leake 1994) depend on their mycorrhizal fungus not only for water and nutrients but also for 100% of their carbon supply. This essential dependency should increase the evolutionary pressure towards a well-organized mycorrhiza in MHP. This holds in particular for MHP with limited surface of the fungi-receiving organs, resulting in reduced fungus colonization probability (the “mycoheterotroph’s dilemma”; see Imhof 2010a). Indeed, most MHP have mycorrhizal colonization patterns that achieve a sustained use of the fungus by the plant even with few colonization events (e.g., Gentianaceae, Imhof 1997; Triuridaceae, Imhof 1998; Burmanniaceae, Imhof 2001). This is realized by anatomical compartmentalization: tissues where hyphae degrade are distinct from those in which hyphae remain intact. In addition, these tissue compartments often determine the shape of the colonizing hyphae, often indicating the service they provide, which can be conveyance, distribution, storage, and finally the digestion of fungal material.

**Figure 1:**
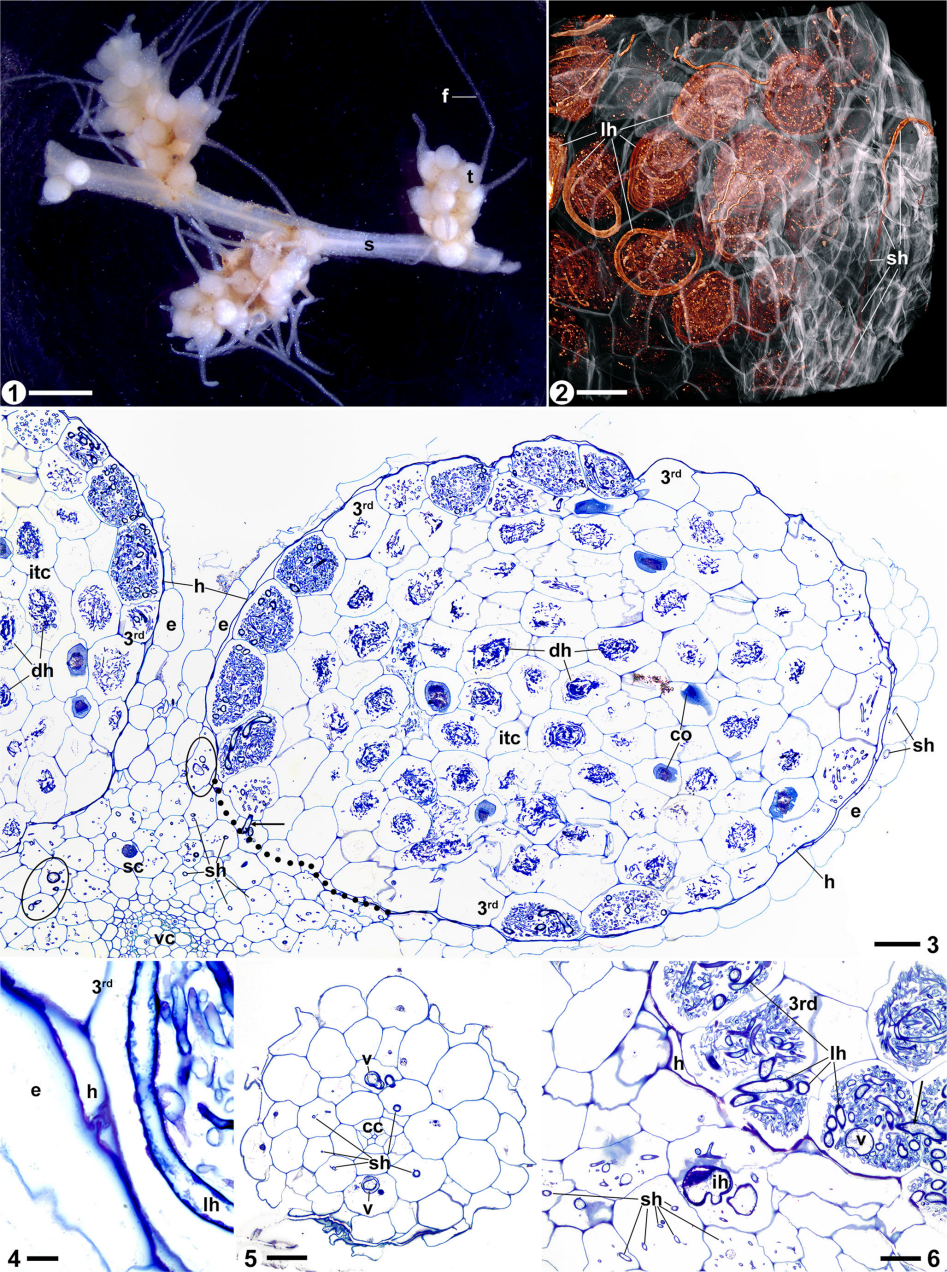

The genus *Afrothismia* (Thismiaceae, after Merckx et al. 2006, 2013 and Stevens 2001 onwards; Thismieae in Burmanniaceae after APG 2009) is of African origin and includes only achlorophyllous MHP not larger than 10 cm in total length (Merckx et al. 2013). To date, 15 *Afrothismia* species have been described; *A. winkleri* comprises two varieties (Imhof 2010b onwards). All *Afrothismia* spp. closely aggregate their roots on rhizome-like shoots resulting in root clusters. Additional root clusters develop in the inflorescence at the pedicel bases of the monochasial cyme (see, e.g., Imhof and Sainge 2008 and Fig. 7), or on side shoots along the main axis (see Fig. 1). The morphology of a single root is remarkable, having a tuber-like base (in the following called tubercle) and a filiform extension of different lengths depending on the species. In *A. gesnerioides* (Maas-van de Kamer 2003), *A. baerae* (Cheek 2003), and *A. kupensis* (Cheek et al. 2019), the filiform part is very short, and only these two species have their root-rhizome systems subterraneous. All other *Afrothismia* spp. cling superficially to leaf litter, rotten wood, or the bare soil surface with their extended filiform root parts (Imhof et al. 2013). In *A. saingei* (Franke 2004), the root-shoot combination harbors one of the most complex mycorrhizal colonization patterns described to date. It shows four different hyphal shapes (straight, looped, inflated coils, degenerating coils) in six separate tissue compartments (filiform root, root epidermis, third root layer, root cortex parenchyma, shoot cortex at root clusters, shoot cortex apart from root clusters). Interconnections between all hyphal shapes demonstrate that they belong to the same fungus (Imhof 1999a^1^). In contrast, the mycorrhizal pattern in *A. gesnerioides* is comparatively simple, with three hyphal forms in five tissue compartments (Imhof 2006; see Table 1).

**Table 1:** Comparisons among mycorrhizal components in *Afrothismia* spp.

	*Afrothismia gesnerioides* (after Imhof 2006)	*Afrothismia korupensis* (this paper)	*Afrothismia hydra* (this paper)	*Afrothismia saingei* (after Imhof 1999a)
Rooting habit	Subterraneous	Epiterraneous	Epiterraneous	Epiterraneous
Length of filiform root part	Up to 5 mm (Maas-van de Kamer 2003)	Up to 16 mm (Sainge et al. 2005)	Up to 50 mm (Sainge and Franke 2005)	Up to 56 mm (Franke 2004)
Hyphae in root epidermis and in filiform root part	Straight, persistent	Straight, persistent	Straight, persistent	Straight, persistent
Hyphae in shoots between the root agglomerations	Straight and persistent in outer cortex	Straight and persistent throughout the cortex and longitudinal ridges	Straight and persistent in outer cortex and longitudinal ridges	Straight and persistent in outer cortex and longitudinal ridges
Hyphae in shoot cortex where roots agglomerate	Straight and persistent, in outer cortex	Straight throughout as well as coiled and inflated (extent: +) in outer cortex, persistent	Straight as well as coiled and inflated (extent: ++), persistent, in outer cortex	Coiled and inflated (extent: +++), persistent, throughout the cortex
Hyphae in the third tubercle layer	Irregularly coiled, persistent	Coils with loops, persistent	Coils with loops, persistent	Persistent coils with distinct loops in a spiral line around the inner cortex, degrading irregular coils in the other cells of the third layer
Inner tubercle cortex	Degrading irregular coils	Degrading irregular coils	Degrading irregular coils	Degrading irregular coils
Starch deposits	Extensively in inner shoot cortex, and inner tubercle cortex without fungal colonization	None	Present in outer shoot cortex, and inner cortex of young tubercles	None

The classical evidence for evolution is traits that appear in transitional series within a group of related organisms. This is most obvious in external morphology (exemplarily shown in Araceae by Engler 1884, or monocot cotyledon by Tillich 1992), but it should also hold for any trait that affects an organism’s fitness. The molecular phylogeny by Merckx and Bidartondo (2008) found *A. gesnerioides* to be ancestral to those *Afrothismia* spp. with longer root extensions and epiterrestrial habit. In searching for linking structures between the considerable morpho-anatomical differences of the two mycorrhizal colonization patterns in *A. gesnerioides* and *A. saingei*, we chose *Afrothismia hydra* (Sainge and Franke 2005) and *A. korupensis* (Sainge et al. 2005), included in the phylogeny by Merckx and Bidartondo (2008), for first anatomical investigations. Moreover, morpho-anatomical details may be helpful for discussion of the familial affiliation of *Afrothismia*, cautiously raised by Merckx et al. (2009).

## Materials and methods

During an expedition to Cameroon, following an invitation from Dr. George B. Chuyong (University of Buea, Cameroon), and under the guidance of Vincent Merckx (Leiden, The Netherlands), a flowering specimen of *Afrothismia hydra* Sainge and Franke (Imhof and Sainge no. 188) and a few fragments of the shoot/root complex of *Afrothismia korupensis* Sainge and Franke presumably shortly after flowering (Imhof and Sainge s.n.) were collected from Korup National Park near Chimpanzee Camp (5°3′50.20′′ N/8°51′25.23′′ E, c. 136 m above sea level) on the 9th of September 2006, fixed in FPA (37% formalin:propionic acid:50% ethanol at a ratio of 0.5:0.5:9). After 2 weeks of fixation, the specimens were transferred into 70% ethanol for permanent storage at the Philipps-University Marburg. The specimens of *Afrothismia saingei* (Wilks 1179) and *Afrothismia gesnerioides* (de Winter 91), kept in spirits and likewise considered in this investigation, also were collected in flowering stage, in January 1986 and April 1996, respectively. Due to the value of the material, only limited specimens could be analyzed and, hence, some variation could have been missed in this study. However, because four different *Afrothismia* spp. from different locations and collecting dates have been considered, which all show the same gross type of colonization pattern, and because the detailed differences between the species are quite specific, inadvertently missed variation should not affect our general conclusion.

The specimens were investigated for their external morphology, photographed with a Moticam 2300 digital camera device (Motic) mounted on a Leica S6D stereo microscope (Leica Instruments), and then prepared for anatomical studies. After dehydration in an ascending ethanol series, roots and root/shoot system were embedded in Unicryl™ (British Biocell Int.). Serial sections of 3–4 μm were made (Leica 2065 Supercut, glass knives prepared by LKB 2078 Histo Knifemaker, LKB Produkter AB, Research Instruments), stained with toluidine blue (1 g toluidine blue O + 1 g sodium tetraborate in 100 mL distilled H_2_O, after Harris in Krause 1927), and mounted in Corbit-Balsam. Histological tests were performed on paraffin-embedded material (Merck, melting point 56–58 °C) sectioned with a Leitz 1512 hand microtome (Leitz) and deparaffinated with xylol. Suberin was tested using the Oil Red O supersaturated isopropanol technique diluted to 60% with distilled water after Lillie (1944), starch was visualized with the iodine test, and lignin was detected by phloroglucine/HCl (Jensen 1962).

Anatomical investigations were performed using a Leitz DMRB-Microscope (Leica Instruments), equipped with a Moticam 2300 digital camera.

Samples for confocal laser scanning microscopy (CLSM) were prepared after the protocol explained in Rath et al. (2014), using Calcofluor White M2R (a non-specific stain that enhances autofluorescence) and WGA (wheat germ agglutinine) conjugated with Alexa Fluor® 633 (fungus specific) as fluorochromes. With a Leica TCX SP5 confocal laser scanning microscope (Leica Instruments), the samples were excited using an UV-Laser (405 nm, for Calcofluor) and a HeNe laser (633 nm, for Alexa Fluor®). The resulting image stacks were analyzed and visualized with AMIRA® (FEI® Visualization Sciences Group, Düsseldorf) and Leica Confocal Software (LCS, Leica Microsystems).

## Results

The anatomy of roots and shoots in *Afrothismia hydra* and *A. korupensis* is similar. The rhizome-like shoots (Figs. 1, 7, and 8), measuring 1.5–2.5 mm in cross section, comprise a pith of parenchymatic cells, surrounded by vascular strands of weekly lignified xylem elements. Phloem elements are not obvious, if present at all. The cortex consists of an endodermis with thicker cell walls than those of the parenchyma (less pronounced in *A. korupensis*) and several uniform layers of cortex parenchyma (Fig. 3). The epidermis is not anatomically distinct except for its cells being slightly smaller. Shoot segments without roots bear slight longitudinal ridges.

The root tubercles of *Afrothismia hydra* (Fig. 7) are 1.3–1.6-mm long and 0.9–1.1-mm wide, and the filiform part can be up to 5-cm long and only about 0.25-mm wide. The tubercles of *A. korupensis* (Fig. 1) tend to be slightly bigger (1.3–1.8-mm long, up to 1.2-mm wide), but the filiform part measured only up to 12 mm in length (0.25-mm wide). The entire root has a tender strand of vascular tissue with only one or two xylem vessels (Figs. 5 and 11). An endodermis could not be detected. In the filiform part, the vascular tissue is surrounded by few layers of parenchymatic tissue and an indistinct epidermis (Fig. 5). In contrast, the tubercle cortex comprises more cell layers and more voluminous cells around the vascular tissue and has a suberized hypodermis separating the cortex parenchyma and epidermis (Figs. 3 and 12). The hypodermis, except for young tubercles and where it merges with the shoot cortex, is largely collapsed and thus forms a double-walled barrier between the epidermis and cortex parenchyma (Figs. 4, 6, and 10). The suberization of the hypodermal cell walls ceases at the root-shoot interface (Figs. 3 and 12, dotted lines). Calcium oxalate crystals (raphids) can occur in all parenchymatic tissues and we never saw raphids and hyphae within the same cell (Figs. 3 and 12). We found starch deposits only in the shoot cortex and the young tubercle cortex of *A. hydra* (Fig. 12).

**Figure 2:**
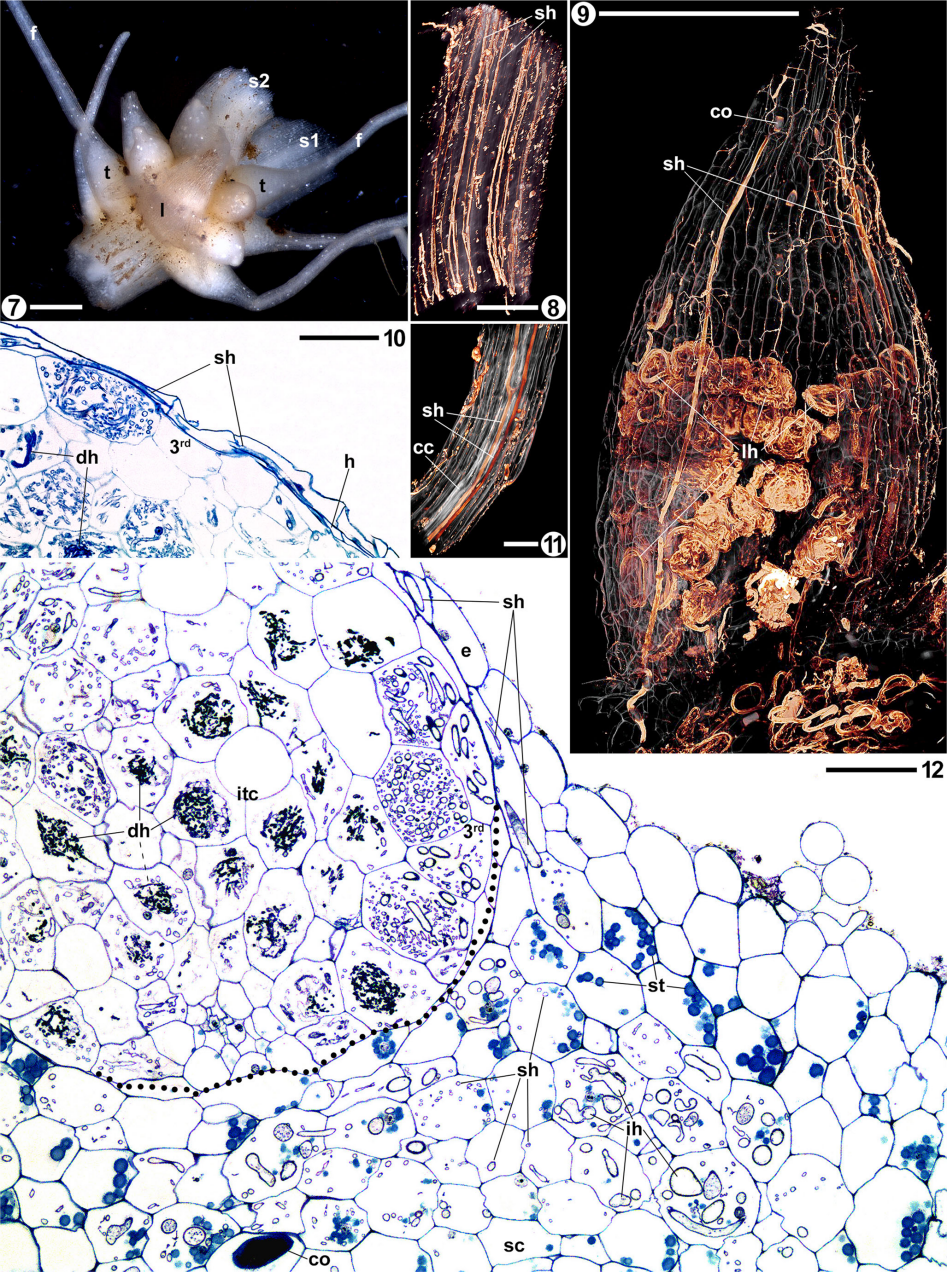

The exclusively intracellular mycorrhizal fungus attains different shapes in separate tissues of *Afrothismia hydra* and *A. korupensis*. The hyphae grow in a straight manner along the axis of the root in the filiform part (Figs. 5 and 11), likewise continuing within the epidermis of the tubercle (Figs. 2, 3, and 9). They never cross the collapsed and suberized hypodermis. Vesicles can occur in the filiform part (Fig. 5).

When the straight hyphae in the tubercle epidermis reach the cortex parenchyma of the shoot, they either maintain the straight mode of growth along the shoot axis (as such interconnecting the spaced root clusters (Fig. 8), vesicles can occur) or swell irregularly and begin to coil (Fig. 3). This swelling and coiling are more pronounced in *A. hydra* (Fig. 12) than in *A. korupensis* (Fig. 3), but are confined in both species to the shoot cortex where roots attach. In contrast to the coils in the inner tubercle cortex (see below), these coils do not degenerate. In *A. hydra*, the inner shoot cortex is free of hyphae; in *A. korupensis*, hyphae can occur throughout the cortex (Fig. 3). Coming from the swollen coils, only one hypha enters the third root layer of the tubercle by crossing the root-shoot interface where the hypodermis has not collapsed and where suberization has ceased (Fig. 3). In the third tubercle layer, the hyphae form loops of thick hyphae in the cells, with smaller, irregularly coiled branches filling the gap in the loop (Figs. 2, 4, 9, and 12). Only thick hyphae pass between cells (Fig. 6). This hyphal mode is kept intact within this layer, gradually expanding the colonization therein towards the tubercle apex, thus encompassing the inner cortex (Fig. 9). Hyphal branches that leave the third layer into the inner cortex parenchyma coil irregularly and, in contrast to the hyphae in all other tissue compartments, degenerate (Figs. 3, 10, and 12). Arbuscules were never seen.

## Discussion

The morphology of root fungi in *Afrothismia* has been interpreted as a complex morphotype of a *Paris*-type arbuscular mycorrhiza (AM, Imhof 1999a, 2006); its identity as an AM fungus was later corroborated by molecular methods (Franke et al. 2006; Merckx and Bidartondo 2008; Merckx et al. 2012). Moreover, the fungi found in *Afrothismia* spp. belong to only one (IN *A. hydra, A. korupensis, A. saingei*) or to three (IN *A. gesnerioides*) virtual taxa from the Glomeraceae (formerly *Glomus* group A, Merckx et al. 2012). *A. gesnerioides* takes permanent advantage of penetrating mycorrhizal hyphae by hyphal forms that seem to accommodate the following demands in distinct root-shoot compartments. Straight and persistent hyphae in the short filiform root part, the tubercle epidermis, and the shoot cortex serve for long distance transport of matter. Coiled and persistent hyphae in the third tubercle layer appear to function for fine-scale distribution around the inner tubercle cortex. Finally, coiled hyphae in the inner tubercle cortex parenchyma degenerate into amorphous clumps. Because at the same time hyphae in all other tissue compartments do not degrade, this phenomenon most probably indicates the digestion of fungal matter for carbon and nutrient uptake. A mostly collapsed hypodermis separates the straight from the coiled hyphae in the epidermis and the cortex of the tubercle (details in Imhof 2006). So far, this is quite similar to the mycorrhizal patterns described here for *A. hydra* and *A. korupensis*. However, in *A. gesnerioides*, the inner shoot cortex and the inner tubercle cortex contain copious starch grains. Indeed, starch deposits also occur in *A. hydra*, albeit to a lesser extent, but starch is entirely missing in *A. korupensis* and *A. saingei* (Imhof 1999a). Converting assimilates into starch for storage is helpful for autotrophs, where assimilation is uncoupled from storage in space or time. In MHP, however, this is not the case. The carbon source, namely the fungus, is always present in the mycorrhizal tissues, and, due to the woody autotrophs in a tropical lowland forest as the ultimate carbon source for the fungus, carbon flux should be well buffered. Hence, the fungal hyphae themselves may potentially serve as storage organs, and seen from that perspective, the conversion into starch seems to be an ancestral metabolic detour. Consistent with this view, *A. korupensis* and *A. saingei*, as well as *A. hydra* in an intermediate stage, have omitted starch storage. Significantly, parallel to the reduction of starch deposits, these species show persistent vesicle-like hyphal coils in the rhizome, absent in *A. gesnerioides*, obviously appropriate to store energy-rich compounds. Although this storage is located in the mycorrhizal fungus, it ultimately will be beneficial for the MHP. Because the degradation of hyphal coils, interpreted as reflecting the assimilation of carbon and mineral nutrients from the fungus, happens exclusively in the inner tubercle cortex, it appears adequate that these storage hyphae only occur in shoot segments where tubercles attach, more extensively in *A. hydra* than in *A. korupensi*s. *A. saingei* has maximized this storage strategy: its entire rhizome-like shoot cortex at the root clusters is packed with such inflated, coiled hyphae (Imhof 1999a). We consider this transition to be a measure to reduce metabolic effort and as such to be an improvement of mycorrhizal benefit from the plant’s perspective.

Straight hyphae occur in the shoot cortices as well: straight hyphae alone in *A. gesnerioides* (Imhof 2006), straight hyphae along with inflated coils in *A. hydra* and *A. korupensis*, and in *A. saingei* straight hyphae only occur in shoot segments apart from root clusters (Imhof 1999a). These straight hyphae interconnect the numerous root clusters along the entire plant up to the inflorescence and, like the inflated coils, do not degenerate. The differences in this regard between the four *Afrothismia* species reflect a transitional change towards more functional complexity and strict partitioning of conveyance (straight hyphae) and storage purposes (inflated hyphal coils).

*Afrothismia gesnerioides* has irregular coils of thin hyphae in the third tubercle layer, whereas those in *A. hydra* and *A. korupensis* are differentiated as looped thicker hyphae and irregularly coiled thinner branches. All these coils remain intact and encase the inner tubercle cortex in a collar-like manner in order to send hyphal branches into the inner cortex parenchyma, where they degenerate. Although the purpose of these loops is unknown, they do not appear to be accidental, because they also develop much more distinctly in *A. saingei* (Imhof 1999a). Furthermore, in *A. saingei*, they do not inhabit the complete third tubercle layer, but are restricted to a spiral line of cells around the inner tubercle cortex. Cells of the third tubercle layer that are not colonized by the looped coils contain irregular coils which degenerate (Imhof 1999a). Keeping in mind that only the inner tubercle parenchyma is able to degrade hyphal coils and, therefore, most probably is essential for the carbon input for *Afrothismia* spp., hyphae must reach all its cells in order for it to use its full digesting capacity. Because the hyphae-degrading forces are likely to retard hyphal growth, the hyphae should use as few cell passages as possible to reach the tubercle center. From a geometric perspective, the spiral line in *A. saingei*, compared with the collar-like variants in *A. gesnerioides*, *A. hydra*, and *A. korupensis*, is the more parsimonious option to always keep the shortest distance to the center and to any other cells of the inner tubercle parenchyma. As a result, in *A. saingei*, fewer cells are necessary to keep hyphae intact for this fine-scale distribution purpose, and more cells can contribute to the acquisition of carbon and nutrients through degradation of hyphae. We consider this a further advanced trait, improving the benefit from the mycorrhizal fungus by a parsimonious strategy.

The loops of hyphae in the third root layer of three *Afrothismia* species are too peculiar to be ignored. In order to reduce the extent, and thus the costs, of the fine-scale distribution in the third tubercle layer, a cellular recognition process is mandatory for determining which coils must remain intact and which can be digested. The loops might play a role in this recognition process. While in *A. gesnerioides* the fungal coils are alike in all cells of the cortex, the looped coils in *A. hydra* and *A. korupensis* offer a structural means of differentiation among coils. On this basis, evolution could have selected the most parsimonious strategy of fine-scale distribution found in *A. saingei*. This hypothesis might also help to explain why hyphal coils within the same root layer degrade whereas others persist.

Although morphology and anatomy of the root-shoot complexes in *Afrothismia* species are similar, the mycorrhizal colonization patterns differ significantly. Because hyphal structures in mycorrhizas are predominantly determined by the plant (based on a prepenetration apparatus; see Genre et al. 2005, 2008), and due to the gradual changes found (summarized in Table 1), the different colonization patterns may be viewed as an evolutionary progression within the genus to improve benefit from the fungus. From this perspective, the basal position of *A. gesnerioides* within its genus based on the molecular phylogenies published in Merckx and Bidartondo (2008) and Merckx et al. (2009) is reflected by the ample starch depositions, the diffuse coils, and the lack of storage hyphae. The retention of starch depositions in *A. hydra* therefore might suggest it to be less derived than *A. korupensis* which is without starch grains. On the other hand, the length of the filiform root parts and the extent of the inflated coils in the shoot cortex suggest that *A. hydra* may be the more advanced species. It is the latter view which is corroborated by molecular inferences in Merckx and Bidartondo (2008) and Merckx et al. (2009) who deem *A. korupensis* to be basal to *A. hydra*. Although *A. saingei* is not included in the phylogeny by Merckx and Bidartondo (2008), we suspect it to be the most advanced species for the following reasons: it has the most extensive occurrence of inflated coils in the root-bearing shoot cortex, the longest filiform root parts, and the most distinguished hyphal loops in the third layer (already visible externally; see Imhof 1999a) which in addition are organized in a spiral line of cells, and the straight hyphae in shoot cortices are restricted to those parts without root agglomerations. Another indication for the progressive character of *A. saingei* may be the state of the hypodermis, which probably is functionally important in all *Afrothismia* spp. as a barrier to prevent the fungus from entering the tubercle cortex directly. In *A. korupensis* and *A. hydra*, remnants of radial cell walls still can be found (see Figs. 4, 6, and 10), whereas in A*. saingei*, the hypodermis is collapsed to a degree that it became overlooked altogether in Imhof (1999a, the third tubercle layer was interpreted as the hypodermis).

A comparable mycorrhizal progression, albeit less complex, was detected in the mycoheterotrophic genus *Voyria* (Gentianaceae). In *V. truncata*, the benefit from a mycorrhizal colonization is not sustained, because penetrating hyphae become degraded right after a couple of cell passages in the root cortex (Imhof and Weber 1997). Compartmentalization of the root cortex in *V. tenella* enables the survival of hyphae in the inner cortex layers and allows a permanent benefit from a fungal colonization event (Imhof 1997). An intermediate colonization pattern was found in *V. aphylla*. By contrast with *Afrothismia*, the progression in *Voyria* is accompanied by a massive reduction of the root system: from runner-like roots up to several meters long in *V. truncata* to little star-like root clumps hardly bigger in circumference than the nail of a little finger in *V. tenella* (Imhof et al. 1994). *V. aphylla* is intermediate to this respect, also (Imhof 1999b). The familial affiliation is undisputed in the case of *Voyria*, which belongs to Gentianaceae since its first description by Jacquin (1763, as *Gentiana aphylla*). *Afrothismia* has been attributed to the tribe Thismieae (Miers 1847) in Burmanniaceae by its author (Engler 1905 as *Thismia winkleri*, transferred to *Afrothismia* by Schlechter 1906), but molecular characterizations by Merckx et al. (2006) revealed the monophyly of Thismieae being sister to *Tacca* (mostly considered to be Dioscoreaceae), whereas Burmannieae are sister to the other Dioscoreaceae, making Burmanniaceae as well as Dioscoreaceae polyphyletic. Accordingly, most recent publications see Thismiaceae (Agardh 1858), including *Afrothismia*, as a separate family (e.g., Lam et al. 2018; Cheek et al. 2018; Sochor et al. 2018; Chantanaorrapint and Suddee 2018; Nishioka et al. 2018; Stevens 2001 onwards). However, some inference methods calculated by Merckx et al. (2009) place *Afrothismia* spp. in a separate clade as sister to the pair of *Tacca* and the remaining Thismiaceae genera (Merckx et al. 2009) resulting in a paraphyletic Thismiaceae. Early investigations on the mycorrhizal structures of *Thismia* spp. describe a disparate and much less sophisticated colonization pattern (Groom 1895; Janse 1896; Meyer 1909; Pfeiffer 1914; Goebel and Süssenguth 1924; Coleman 1936; McLennan 1958; Campbell 1968) compared with those in *Afrothismia* spp. discussed in this paper. Hence, molecular data, classical morphological differences (listed in Merckx et al. 2009), and the distinctive mycorrhizal progression presented here argue for a deeper distinction between Thismiaceae and *Afrothismia* than is currently accepted.

## Footnotes

1 The specimen (Wilks 1179) investigated in Imhof (1999a), labeled as *Afrothismia winkleri*, turned out to be a new species, later described by Franke (2004) as *Afrothismia saingei* (Maas-van de Kamer and Maas 2010).
